# Ultrasound-Assisted Aqueous Extraction of Biocompounds from Orange Byproduct: Experimental Kinetics and Modeling

**DOI:** 10.3390/antiox9040352

**Published:** 2020-04-23

**Authors:** Esperanza Dalmau, Carmen Rosselló, Valeria Eim, Cristina Ratti, Susana Simal

**Affiliations:** 1 Department of Chemistry, University of the Balearic Islands, Ctra. Valldemossa km. 7.5, 07122 Palma de Mallorca, Spain; esperanza.dalmau@uib.es (E.D.);; 2 Department of Soils Science and Agri-Food Engineering, Institute of Nutrition and Functional Foods (INAF), Laval University, Québec City, QC G1V 0A6, Canada

**Keywords:** orange byproduct, ultrasound-assisted extraction, antioxidant compounds, Weibull model

## Abstract

Orange byproduct (flavedo and albedo) from juice extraction, was used as raw material for this study. Kinetics of total phenolic and total flavonoid contents and antioxidant activity was experimentally determined during both conventional (agitation at 80 rpm) and ultrasound assisted (at 520 and 790 W/L) aqueous extraction from orange byproduct at 5, 15, and 25 °C. An extraction mathematical model was also developed. Significant increase of biocompounds extraction yields was observed as temperature and acoustic power density increased. Ultrasound assistance allowed higher yields at lower temperatures and shorter times. Yields of total phenolic and total flavonoid contents and antioxidant activity obtained with ultrasound extraction (790 W/L, 25 °C, 3 min) were 29%, 39%, and 197% higher, respectively, than those obtained by conventional extraction. The extraction kinetics curves were properly represented by the Weibull model for both conventional and acoustic extraction (mean relative error lower than 5%). Naringin, neohesperidin, and hesperidin were the main phenolic compounds found in the extracts, followed by ferulic, sinapic, and cuomaric acids. Neohesperidin, hesperidin, coumaric acid, and sinapic acid presented the highest yields, especially when extraction was assisted by ultrasound. Meanwhile, naringin and ferulic acid were extracted in a lesser extent, most likely due to their lipophilic character.

## 1. Introduction

Spain produces more than one million tons/year of oranges, being the largest producer of this fruit worldwide. The industrial use of a part of these oranges, especially for the production of juice, results in the accumulation of high amounts of byproducts (37,800 tons/year [1]), consisting mainly of peel and flesh, which account for about half of the fruit weight. In Spain, the waste from orange juice production is typically used as animal feed or discarded. However, this byproduct is rich in numerous biologically active compounds such as phenolic acids and flavonoids [2]. Currently, the extraction of these bioactive compounds from the orange byproducts has had considerable scientific interest to use them as natural source of antioxidants, especially for food formulation to prevent the oxidation of lipids [2]. Indeed, in recent years, a lot of research has focused on plants and their by-products to extract natural and low-cost antioxidants that can replace synthetic additives such as butylated hydroxyanisole (BHA) and butylated hydroxytoluene (BHT), which might be liver-damaging, carcinogenic, and more toxic in general [3].

Scientists have studied various processes for extracting compounds with bioactive activity from citrus peels. These treatments include conventional solvent extraction, supercritical CO_2_ extraction, pressurized fluid extraction, microwave-assisted extraction, and enzyme-assisted extraction [4]. Over the past few decades, intensive research in this field has enabled the development of more efficient and environmentally friendly extraction methods. Among the main characteristics of the ultrasound assisted extraction (UAE), it is worth to mention the lower extraction times and temperatures, and higher extraction yields [5], in comparison to conventional extraction. The mechanism for ultrasonic enhancement is mainly attributed to behaviors of the bubbles of cavitation upon the propagation of the acoustic waves [6]. Collapse of bubbles can cause chemical, physical, and mechanical effects, which result in the disruption of biological cell walls to facilitate the release of extractable compounds and enhance mass transport of solvent from the continuous phase into the plant cells [7]. Optimization of ultrasonic-assisted extraction has been described to extract total phenolic content from olives leaves (var. Serrana) [5], mineral elements, sugars and carotenoids from apple juice [8], and total phenolic and total monomeric anthocyanin content from eggplant peel (*Salanum melongena L.*) [9]. In these works, methanol came up as a suitable extraction solvent to reach good yields of the bioactive compounds. However, environmentally benign and non-toxic food grade organic solvents are recommended for extraction purposes by both the directive 2009/32/EC of the European Parliament and the US Food and Drug Administration [10].

In any case, extraction rate of the target compounds depends on the effect of the different operating experimental conditions, there being a renewed interest in optimizing all these process variables to the best utilization of energy, time, raw material and/or solvent [11]. From a food engineering point of view, mathematical modeling has been widely used for this purpose in the last decades, since it provides a quick and inexpensive determination of the effects of the experimental conditions on the outcome of the extraction process. Analyses of the kinetic parameters and their dependence on the operating conditions have led to a better understanding of the mass transfer mechanisms, facilitating the design, simulation, optimization, and control of industrial processes [5,11]. Kinetic models of solid–liquid extraction may be based on either phenomenological or empirical equations. The latter, formulated from experimental data, attempt to determine the relevant variables of the process and the relationships among them. Although they do not physically explain the extraction process, the empirical models may indeed be very useful for certain industrial applications or for simplifying the study of complex systems, hard to formulate and/or solve by phenomenological models [12]. Such is the case of the extraction of bioactive compounds from plant materials, due to the heterogeneity of the samples and the multicomponent complexity of the mass transfer mechanisms.

A literature research did not provide any reference about earlier reports on the modeling of the UAE of phenolic compounds using mathematical equations and food grade solvent such as water. Therefore, the main aim of the present research was to investigate the effects of both temperature and application (or not) of power ultrasound on the extraction rate of polyphenolic compounds and antioxidant activity from orange byproducts. Furthermore, this study aimed at proposing an empirical model to simulate the extraction kinetics, using either mechanical agitation or ultrasound application, which properly relates changes in phenolic compounds content and antioxidant activity of the aqueous extracts to the ultrasonic power density, temperature, and extraction time.

## 2. Materials and Methods

### 2.1. Chemicals and Plant Material

Total polyphenol content (TPC), total flavonoid content (TFC), and antioxidant activity (AA) reagents were purchased from Sigma-Aldrich (St. Louis, MO, USA). Neohesperidin, naringin, coumaric acid and ferulic acid were bought from Fisher (Madrid, Spain), while Hesperidin and sinapic acid, from Sigma-Aldrich (St. Louis, MO, USA). All reagents were analytical grade or higher.

Oranges (var. Navelina) were purchased from a local market in 2019, from which those that had a soluble solid content of 11.0 ± 0.5 °Bx, were selected. After the juice extraction, the byproducts composed of both flavedo and albedo, were used as raw material for the study. In order to obtain a homogeneous and stable byproduct, the raw material was first steamed for 5 min; then freeze-dried (Telstar LyoQuest, Barcelona, Spain) at a temperature of −50 °C and a vacuum pressure of 30 Pa, until a final moisture content of 6.93 ± 0.07 kg H_2_O/100 kg dm. The freeze-dried byproduct was ground (0.355–0.710 mm screen) with a blade mixer (A10, Analysenmühle, IKA, Staufen, Germany) and stored at 4 °C until further use. This final byproduct was analyzed to determine its TPC, TFC, AA (by using the ABTS (2.2′-azino-bis(3-ethylbenzothiazoline-6-sulfonic acid), FRAP (ferric reducing antioxidant power), and CUPRAC (cupric reducing antioxidant capacity) assays), and antioxidant compounds by the high-performance liquid chromatography (HPLC) method.

### 2.2. Conventional and Acoustic Extraction Process

The extraction process was carried out following the extraction method reported by González-Centeno et al. [11]. Water was used as extraction solvent at a solid/solvent ratio of 1:60 (*w/v*, g/mL) with a total extraction volume of 200 mL. Extractions were performed in a vessel with a double glass layer to control temperature throughout the process. For this purpose, a peristaltic pump (VitaTech 600, Vitakraft, Bremen, Germany) recirculated a 50% (*v/v*) ethylene glycol solution from the cooling reservoir, equipped with a chiller unit (Frigedor, J.P. Selecta, Barcelona, Spain), through the jacketed extraction vessel.

Both conventional and ultrasound-assisted extraction processes at three different temperatures (5.0 ± 0.3 °C, 15 ± 1 °C and 25 ± 2 °C) were carried out. Conventional extraction (CE) was conducted with mechanical agitation (80 rpm) by using a stirrer (RZR 2021, Heidolph, Schwabach, Germany) equipped with a 4-blade propeller (50 mm diameter), placed at the central point of the total extraction volume. For the extractions with acoustic assistance (UAE), the mechanical stirring was replaced by an ultrasonic device consisting of a probe system (UP400S, Hielscher Ultrasound Technology, Teltow, Germany) immersed into the extraction solution and working in cycles of 0.5 s. Thus, UAE was carried out by using two different probes, of 40 mm (UAE1) and 14 mm (UAE2), in order to test different acoustic power densities. Three replicates were carried out of each experiment.

Extract aliquots (2 mL) were taken at 1, 2, 3, 4, 5, 6, 8, 10, and 30 min of extraction, filtered (RC-membrane, 0.45 µm, Sartorius Stedim Biotech GmbH, Göttingen, Germany) and stored in eppendorfs at −20 °C until the analyses were performed. These samples were analyzed to determine their TPC, TFC, AA (by using the ABTS, FRAP, and CUPRAC assays) and antioxidant compounds by HPLC method.

### 2.3. Characterization of the Ultrasonic Power Density

Before the ultrasound extraction experiments, a calorimetric approach was used out to measure the effective ultrasonic power transferred into the medium, in accordance with the procedure described by Kimura et al. [13] done in the absence of sample and without thermostating the system. Briefly, the temperature of the solvent was logged every 1 s for the first 5 min of ultrasound application by using two K-type thermocouple probes connected to a data acquisition equipment HP 34970A Data Logger (Hewlett-Packard, Barcelona, Spain). The experimental temperature rise, caused by the dissipation of the acoustic waves, was used to calculate the effective ultrasonic power applied (P) as expressed in Equation (1). The ultrasonic power was measured in triplicate for each acoustic condition:

(1) $$P=m\cdot C_{p}\cdot \frac{dT}{dt}$$

The acoustic power density (W/L) was calculated as the ratio between the ultrasonic power applied (P) and the total extraction volume. C_p_ is the specific heat of water in J/kg °C.

### 2.4. Total Polyphenol Content (TPC), Total Flavonoid Content (TFC) ans Antioxidant Activity (AA)

Total polyphenol content (TPC) was determined by means of the Folin-Ciocalteu assay according to Eim et al. [14] and the total flavonoid content (TFC), from the Aluminum chloride assays according to Leontowicz et al. [15]. The antioxidant activity (AA) was determined by using the ABTS (2 2′-azino-bis(3-ethylbenzothiazoline-6-sulfonic acid), FRAP (ferric reducing antioxidant power), and CUPRAC (cupric reducing antioxidant capacity) assays according to González-Centeno et al. [16]. Methanol extracts from raw material were prepared according to the methodology described by Eim et al. [14] with minor modifications. Samples were weighed (~1.5 g), and 20 mL of methanol (MeOH) extraction solvent was added. Mixtures were homogenized using an Ultra-Turrax T25 Digital (IKA, Staufen, Germany) at 13,000 rpm for 1 min at 4 °C, and these solutions were refrigerated overnight. The mixtures were then centrifuged at 4000 rpm for 10 min followed by filtration to obtain the methanol extract. The extracts were refrigerated at 4 °C until analysis.

In all assays, absorbance measurements were carried out at 25 °C in an UV/Vis/NIR spectrophotometer (MultiSkan Spectrum, Thermo Scientific, Vantaa, Finland) and correlated with standard curves. TPC, TFC, and antioxidant activity (ABTS, CUPRAC and FRAP assays) were first measured for the initial orange byproduct before extraction (C_max_).

The extraction yield (Y) of TPC, TFC, and AA (using the ABTS, CUPRAC and FRAP assays) at each extraction time, was expressed as indicated in Equation (2).

(2) $$Y=\frac{C}{C_{max}}\cdot 100$$

where C_max_ is the concentration in the initial byproduct and C is the concentration of the extract at each extraction time referred to the initial dry matter of the sample. 

The TPC was expressed as mg gallic acid equivalent (GAE)/100 g dm. The TFC was expressed as mg catechin/100 g dm. The AA was expressed as mg Trolox/100 g dm. All analyses were performed in triplicate.

### 2.5. Mathematical Modeling

A mathematical model was proposed with the aim of establishing a methodology to analyze the mass transfer process during the conventional and ultrasound-assisted extraction of bioactive compounds from orange juice byproduct. The proposed model to mathematically describe the extraction kinetics was the Weibull model (Equation (3)).

(3) $$\frac{C-C_{eq}}{C_{o}-C_{eq}}=e^{\left[-{\left(\frac{t}{\alpha }\right)}^{\beta }\right]}$$

In this model (Equation (3)), C_eq_ is the equilibrium concentration that was assumed to be equal to the experimental value after 30 min of extraction [11], and C_o_ is the initial concentration in the extract (equal to zero). The α parameter of the Weibull model can be related to the inverse of the change rate. As such, a lower α indicates faster rate of change of a given quantity. In general, the parameter α is observed to be dependent on the temperature according to an Arrhenius type equation (Equation (4)) [11]:

(4) $$\alpha =\alpha_{o}\cdot e^{\left(-\frac{E_{a}}{RT}\right)}$$

The shape parameter β represents a behavior index of material during the extraction process [17]. When β is equal to 1, the model corresponds to a first order kinetic with a constant input rate [14]. However, when β has a value above or below 1, this parameter denotes the concavity (increasing change rate over time) or convexity (decreasing change rate over time) of the curve, respectively [18].

Taking into account Equations (2)–(4), the proposed model could be written as follows (Equation (5)):

(5) $$Y=Y_{eq}\left[1-e^{\left[-{\left[\frac{t}{\alpha_{o}\cdot e^{\left(-\frac{E_{a}}{RT}\right)}}\right]}^{\beta }\right]}\right]$$

For each determination (TPC, TFC, and AA), all the experimental data obtained at different extraction temperatures and times were simultaneously used to carry out the identification of the Weibull model parameters (α_o_, E_a_ and β of Equation (5)) for each set of experiments (CE, UAE1, and UAE2). The ‘fitnlm’ function of the optimization toolbox of Matlab R2014a (The MathWorks Inc., Natick, MA, USA), which estimates the coefficients of a nonlinear regression function and the residuals using least squares, was used to identify the model parameters. To determine the 95% confidence intervals (CI) and the standard error of the estimated parameters (SE), the ‘coefCI’ function and the covariance matrix were used, respectively.

### 2.6. High-Performance Liquid Chromatography (HPLC) Analysis

The polyphenol compounds were determined by HPLC, as described by M’hiri et al. [19]*,* with some modifications. The quantitative analysis was performed by using an HPLC analytical system (Waters, Milford MA, USA) consisting of a Waters 600E pump, a Waters 2966 photodiode array detector and a Waters 717 plus autosampler controlled by software (Empower). For each analysis, 10 µL of extract was injected on reverse-phase C18 column (150 × 3.9 mm, 5 µm particle size, Symmetry, Brookfield, WI, USA). The mobile phase consisted of solvent A, water-acetic acid (3%) and solvent B, methanol-acetic acid (3%). A gradient program was carried out as follows: 5 min, 10% B; 30 min, 100% B; 45 min, 100% B; 47 min, 10% B. The flow rate was 1 mL/min, and the temperature of the column oven was 40 °C. Flavanones (naringin, hesperidin, and neohesperidin) and phenolic acids (ferulic acid, sinapic acid, and p-coumaric acid) were detected at wavelengths of 280 and 320 nm, respectively. Quantification was carried out by using the external standard method and the final concentrations were calculated in mg/100 g dm. The linearity of naringin, hesperidin, neohesperidin, ferulic acid, sinapic acid, and p-coumaric acid calibration curve was confirmed by the coefficients of determination (*r^2^* = 0.988; 0.977; 0.973; 0.978; 0.992 and 0.986, respectively). The limits of detection of naringin, hesperidin, neohesperidin, ferulic acid, sinapic acid and p-coumaric acid were of 0.87; 0.32; 0.89; 0.96; 0.87, and 0.45 mg/L, respectively. The limits of quantification of naringin, hesperidin, neohesperidin, ferulic acid, sinapic acid, and p-coumaric acid were of 2.5; 2.0; 3.2; 2.1; 2.2, and 1.9 mg/L, respectively. The retention times of naringin, hesperidin, neohesperidin, ferulic acid, sinapic acid, and p-coumaric acid were of 21.1; 22.4; 23.5; 20.0; 18.2; and 16.8 min, respectively. All analyses were performed in triplicate.

### 2.7. Statistical Analysis

Results are presented as mean values with their corresponding standard deviations. Statistical analyses were carried out following the procedure described by Dalmau et al. [20]. Statistical analyses were performed using R 3.1.0 software (R Core Team, 2017). Parametric ANOVA and Tukey tests were used to evaluate the existence and the degree of the significant differences, respectively. These statistical analyses were replaced by Kruskal-Wallis and pairwise-Wilcox (BH corrected), when data were not normally distributed and/or showed heterogeneity of variances. Differences at *p* < 0.05 were considered significant.

The mean relative error (MRE, Equation (6)), estimated by the comparison of experimental (Y_exp_, %) and simulated (Y_cal_, %) extraction yields, was calculated to statistically evaluate the accuracy of the proposed mathematical model to simulate the extraction kinetics.

(6) $$MRE=\frac{100}{n}\cdot \overset{n}{\underset{i=1}{\sum }}\left(\frac{\left|Y_{i}{}_{exp}-Y_{i}{}_{cal}\right|}{Y_{i}{}_{exp}}\right)$$

## 3. Results and Discussion

A calorimetric method described before was used to determine the acoustic power density (W/L) in the system used in this study, for the UAE1 and UAE2 experiments. By dividing the ultrasonic power by the total extraction volume, the obtained acoustic densities were of 520 ± 16 W/L for the 40 mm probe (UAE1) and of 790 ± 19 W/L for the 14 mm (UAE2) probe.

The initial TPC, TFC and the AA of the byproduct are shown in Table 1. These results are in agreement with the findings of Wang et al. [21] for TPC (1650 ± 50 mg GAE/100 g dm), with Sade-Omaba et al. [22] for TFC (4.2 ± 0.02 mg QE/g dm), with Zhang et al. [23] and Gironés-Vilaplana et al. [24] for AA by ABTS and FRAP, respectively (2670 ± 200 and 1480 ± 300 mg Trolox/100 g dm, respectively).

### 3.1. Kinetics of Biocompounds Extraction

The TPC, TFC, and AA (using the ABTS, FRAP, and CUPRAC assays) of extracts, taken at 1, 2, 3, 4, 5, 6, 8, 10, and 30 min of extraction, were measured to determine the kinetics of extraction. Results of the AA obtained by the three proposed methods (ABTS, FRAP, and CUPRAC) were highly, significantly and positively correlated (*r^2^* ≥ 0.86; *p* < 0.05). Therefore, the results of the AA extraction obtained by only one of the three methods (ABTS assay) are presented.

Table 2 shows the equilibrium yields in the extracts, calculated from the extracts concentrations after 30 min of extraction, of TPC, TFC, and AA (ABTS assay) for mechanical agitation (CE at 80 rpm) and acoustically assisted (UAE1 at 520 W/L; UAE2 at 790 W/L) extraction and the observed temperature dependence within the temperature range 5–25 °C. The equilibrium extraction yield of TPC was constant with the temperature but increased when ultrasound was applied ca 11% (UAE1) and 23% (UAE2). However, the equilibrium extraction yield of TFC and AA linearly (*r^2^* > 0.98) increased with the temperature in all cases, but also with the acoustic power density (except at 25 °C for TFC extraction under UAE1 and UAE2 conditions). In the case of the AA equilibrium yield, the effect of the ultrasound was more intense than that of the temperature, increasing from ca 37% in CE experiments to 68% in UAE1 and 92% in UAE2 as average. These important increases in the AA yield may be due to the enhanced solubility of analytes within the extraction solvent and to the positive sum of analytes that previously did not have original antioxidant activity and those that lost their original antioxidant activity [25].

**Figure 1:**
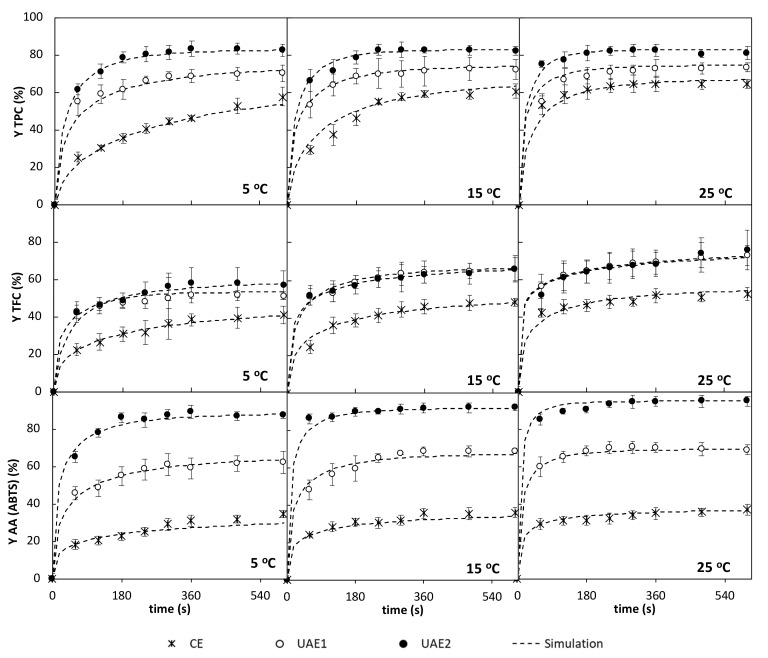
Experimental (average ± deviation) and simulated extraction yield curves of total polyphenol content (TPC), total flavonoid content (TFC), and antioxidant activity (AA, ABTS (2.2′-azino-bis(3-ethylbenzothiazoline-6-sulfonic acid) assay) from orange byproduct at different temperatures (5, 15, and 25 °C) under mechanical agitation (CE at 80 rpm) and acoustically assisted (UAE1 at 520 W/L; UAE2 at 790 W/L) conditions.

The experimental curves of extraction yield of the TPC, TFC, and AA (ABTS assay) from orange byproduct at different temperatures (5, 15, and 25 °C), with mechanical agitation (CE at 80 rpm) and with acoustic assistance (UAE1 at 520 W/L; and UAE2 at 790 W/L), are shown (dots) in Figure 1 (TPC, TFC and AA). It can be observed in Figure 1 that most of the TPC extraction took place during the first 200 s of the process, the yields increasing sharply from 0 to 60 s and less pronouncedly from 60 s onwards. The only exception was the experiment at 5 °C under mechanical agitation (CE), in which the yield kept increasing during the 600 s of the experiment. The effect of the temperature was observed mainly in the first part of the curves (from 0 to 200 s). This temperature effect on the extraction efficiency is most likely due to the enhanced solubility of analytes within the extraction solvent and their improved diffusion rate from the solid matrix as temperature increases [26]. This influence was more evident in the experiments carried out under mechanical agitation. These results are in accordance with the results of Jeong et al. [27], for conventional phenolic extraction and antioxidant activity from citrus *unshiu* peel within the temperature range 50–150 °C. However, it is important to point out that usually, temperature has a positive effect when it is not too high (lower than 60–70 °C), as most of bioactive compounds are susceptible to degradation at higher temperatures, reducing the observed extraction rate [28].

An important effect of ultrasound was observed at all studied temperatures, the TPC extraction yield significatively (*p* < 0.05) increasing by applying ultrasound, i.e., 22%, 19%, and 13% increases at 5, 15, and 25 °C, respectively, when comparing yields in UAE1 experiments to those of CE experiments after 600 s of extraction. The increase of acoustic power density from 520 (UAE1) to 790 W/L (UAE2) also promoted increases in the extraction yield of 16 ± 2% within the temperature range 5–25 °C. By comparing results in Table 2 and Figure 1, it can be seen that TPC yields after 5 min of extraction when ultrasound was used, were very similar to those at the considered equilibrium (maximum observed figures). However, more time was needed, even to reach lower figures, when extraction was carried out just by using agitation.

According to the experimental results, even the extraction yield curve of TPC at 25 °C with agitation located under the corresponding curves were at 5 and 15 °C with ultrasound assistance (Figure 1). These phenomena reveal that acoustic assistance to extraction allowed working at lower temperatures than the conventional process without reducing the extraction rate or even improving it. Similar results were previously reported by other authors; Cho et al. [29] observed that only 1 min of acoustic extraction at 25 °C led to the recovery of the same amounts of resveratrol from grape stems as 30 min of conventional extraction at 60 °C; González-Centeno et al. [11] observed that conventional extraction of TPC at 35 and 50 °C from grape pomace did not differ significantly from ultrasound-assisted extractions performed at 20 and 35 °C, respectively.

The effects of both temperature and ultrasound on the TFC extraction yield curves (Figure 1) were different to those observed for the TPC extraction, although, as well as in this case, most of the TFC extraction took place during the first 200 s slightly increasing from this time onwards. The effect of temperature was important, the TFC extraction yield increasing 18% and 27% in CE experiments; 29% and 42% in UAE1 experiments; and 16% and 33% in UAE2 experiments; when temperature increased from 5 to 15 and 25 °C, respectively, after 600 s of process. The effect of the ultrasound application was also important, although the extraction yield curves at 520 and 790 W/L at each temperature were almost coincident, especially at 15 and 25 °C, indicating that the increase of the acoustic power density did not promote increases in the TFC yield extraction within this acoustic power density range. Again, the TFC yield curves at any temperature obtained by using agitation located under those obtained by acoustic assistance extraction.

With regard to the antioxidant activity extraction (Figure 1), most of the AA extraction took place during the first moments of the process, the curves being almost flat after 100 to 200 s of extraction, depending on the temperature. The extraction yield curves at every temperature were very similar among them, within each group of experiments (CE, UAE1, and UAE2), thus, the effect of the extraction temperature was low; however, an important effect of the ultrasound application on the curves was observed, both the initial rate of extraction and the final extraction yield after 600 s significantly (*p* < 0.05) increasing when ultrasound were applied at 520 and 790 W/L. The equilibrium extraction yields (Table 2) only increased by 3.5%, 4.6%, and 7.2% from 5 to 25 °C in CE, UAE1, and UEA2 experiments, respectively; however, they increased ca. 82 ± 1% at 520 W/L of acoustic power density, and 147 ± 4% at 790 W/L of acoustic power density, as average within the 5 to 25 °C temperature range.

Similar results were obtained by Um et al. [30] in the ultrasound-assisted extraction (30 °C, 30 min, and 50% ethanol) of TPC, TFC, and ABTS from rugose rose fruit (50.7–96.7, 15.9–31.9, and 3.1–6.1 mg/g, respectively). Khan et al., [30] observed that TPC obtained by UAE (40 °C, 150 W, 80% ethanol) from orange peel (*Citrus sinesis L.*) during 30 min was 1.4-folds higher than by conventional extraction.

Thus, the yields of TPC, TFC, and AA of the extracts obtained from the CE extraction process performed at 5 °C were the lowest among all the experimental conditions tested (*p* < 0.05). Specifically, after 5 min of extraction at this temperature, TPC was 750 ± 30 mg GAE/100 g dm; TFC was 90 ± 8 mg catechin/100 g dm and AA, 810 ± 80 mg Trolox/100 g dm (ABTS assay), 760 ± 60 mg trolox/100 g dm (CUPRAC assay) and 910 ± 90 mg Trolox/100 g dm (FRAP assay).

Furthermore, as shown in Figure 1, ultrasonic application led to significant reductions of the extraction time needed to obtain aqueous extracts with similar phenolic, flavonoid, and antioxidant characteristics. In particular, the UAE1 acoustic process was ca. 10 times shorter than the CE process to show similar total polyphenolic recovery (~1120 mg GAE/100 g dm). Similar behaviors were observed for flavonoids and antioxidant activity extraction. In line with the present research, Khan et al. [31] observed that the acoustic extraction (25 kHz, 90 W, with an ultrasonic transducer plate) of phenolics compounds from orange peels during 10 min, achieved extracts with the same concentration as the Soxhlet process in 60 min. Ma et al. [32] reported that the total phenolic content of extracts from Satsuma Mandarin peel after 10 min of ultrasonic extraction (8 W at 30 °C) was six times higher than obtained with conventional extraction (without shaking at 40 °C) during 1 h. González-Centeno et al. [11] concluded that the acoustic extraction process (435 W/L) of phenolic content from grape pomace required about 3, 4, and 8 times less time that the conventional process (60 min) to obtain the same total phenolic recovery at 20, 35, and 50 °C, respectively.

Finally, in this study, the highest extraction yields after 10 min of extraction of phenolic (1389 ± 72 mg GAE/100 g dm) and flavonoid (200 ± 19 mg catechin/100 g dm) contents and antioxidant activity (2672 ± 89 mg Trolox/100 g dm (ABTS assay), 2145 ± 55 mg Trolox/100 g dm (CUPRAC assay) and 1687 ± 72 mg Trolox/100 g dm (FRAP assay)) were obtained at 25 °C by UAE at 790 W/L.

### 3.2. Mathematical Modeling

The Weibull model was used to mathematically describe the aqueous conventional (mechanical agitation) and ultrasonically assisted extraction of phenolic and flavonoid compounds and antioxidant activity (ABTS assay) from orange byproduct taking into account the effect of the temperature (from 5 to 25 °C) according to Equation (5).

By using the experimental results of TPC, TFC, and AA (ABTS assay) obtained at different extraction times and temperatures by mechanical agitation (CE) and ultrasound (UAE1 at 520 W/L; UAE2 at 790 W/L) together with the equilibrium extraction yields shown Table 2, and considering the initial extraction yield equal to zero, the α_0_, E_a_ and β parameters were identified for each set of experiments (CE, UAE1 and UAE2).

Results obtained in these identifications are shown in Table 3 together with the confidence intervals and standard errors (SE) associated to each parameter. As it can be seen from the figures shown in Table 3, α exhibited the effects of both the temperature and the ultrasound application on the extraction rate. In general, α decreased with the increase of the temperature and the acoustic power density. Thus, α values for conventional extraction (CE) ranged, for temperatures between 5 and 25 °C, from 279 to 45 s^−1^ for TPC; and from 199 to 49 s^−1^ for TFC; and from 154 to 23 s^−1^ for AA (ABTS assay), thus indicating that the extraction rate increased as temperature increased in all cases. Similar effect of temperature was previously observed in extraction modeling of saponins from quinoa seeds [33], β-carotene from rose hip [17], and total polyphenol content and antioxidant activity from grape pomace [11], by using the Weibull model within the temperature ranges of 20 to 60 °C, 25 to 55 °C, and 20 to 50 °C, respectively.

However, the effect of the temperature was smaller in acoustically assisted extractions, α decreasing from 63 to 18 s^−1^ in UAE1 and from 37 to 19 s^−1^ in UAE2 for TPC extraction; from 48 to 30 s^−1^ in UAE1 and UAE2 for TFC extraction; and from 57 to 15 s^−1^ in UAE1 and from 26 to 8 s^−1^ in UAE2 for AA extraction. The same value of α at each temperature was obtained for extraction of TFC at both acoustic power densities. In overall, α decreased ca. 85–95% by increasing the temperature from 5 to 25 °C and applying ultrasound at 790 W/L instead of mechanical agitation at 80 rpm.

According to the literature, when temperature and ultrasonic power is too high, the extracted compounds might degrade and the global rate of extraction may exhibit a decrease [34,35]. However, the extraction rate in UAE1 and UAE2 experiments at 5 °C was higher than that of the CE experiments at the same temperature, therefore the global extraction was more efficient when applying ultrasound at every temperature (between 5 and 25 °C)—but especially at 5 °C.

As the α parameter was considered a kinetic constant dependent on the temperature according to the Arrhenius equation, the activation energy (E_a_) for the extraction processes, which may be described as the energy barrier that bioactive compounds need to cross in order to be removed from the orange byproduct matrix, was estimated. The E_a_ figure may depend on different factors in the extraction process, such as the food matrix, the target biocompounds, the sample pretreatment, and the solvent used, among others [36]. The estimated E_a_ for CE extractions of TPC, TFC and AA were of 62,900, 48,100, and 66,300 J/mol, respectively. As it was expected, acoustic assistance significantly reduced the E_a_ of biocompounds extraction kinetics. By applying 520 W/L (UAE1) and 790 W/L (UEA2) of acoustic power density, the E_a_ decreased ca. 32% and 63%, respectively, for the extraction of TPC; ca. 68% for the extraction of TFC; and ca. 31% and 38%, respectively, for the AA extraction, in comparison to the mechanical assisted extraction (CE). Similar behavior was observed by Khan et al. [31] for extraction of total phenolic content by UAE (40 °C, 150 W, 80% ethanol) from orange peel (*Citrus sinesis L*.) during 30 min.

As previously mentioned, β, the shape parameter of the Weibull model, is equal to 1 when the kinetic is of first order (constant extraction rate), or lower or higher than 1 when the extraction rate decreases or increases over the time. The shape of the extraction kinetic is much more pronounced as β value move away from the unit. In all sets of experiments (CE, UAE1, and UAE2), β remained constant within the 5–25 °C temperature range and lower than 1 (Table 3), indicating that in all cases, the extraction rate decreased over extraction time. In the case of the TPC and AA extraction, β figures were similar for CE, UAE1, and UAE2 sets. However, for the TFC extraction, β significantly (*p* < 0.05) decreased when ultrasounds were applied, indicating that after the first moments of extraction, the extraction was almost finished. These experimental results were similar to those reported for saponin water extraction with agitation during different times and temperatures from quinoa seeds (β = 0.206–0.305) [33], and for β-carotene extraction with ethylic ether, petroleum ether and n-hexane as solvents, with solid liquid ratio of 1/ 10, 1/20, and 1/40 and different temperatures (25, 35, 45, and 55 °C) from rose hip (β = 0.424–0.644) [17].

To statistically evaluate the accuracy of the proposed model and, therefore, its capacity to simulate the experimental results and to predict variation within the system, the mean relative error (MRE, Equation (6)) was calculated for all the extraction conditions tested by comparing experimental and calculated values. According to the bibliography, the lower the MRE, the better the simulation fit provided by the model [11]. The MRE obtained for the simulation of TPC, TFC and AA extraction kinetics in each set of experiments are shown in Table 4. As it can be observed in this table, the MRE was equal or lower than 5% in all cases, the average MRE being of 3.3 ± 1.6% for TPC extraction; 2.8 ± 0.6% for TFC extraction; and of 3.1 ± 1.7% for the AA extraction.

From Figure 1, and the values of the statistical parameter MRE (Table 4), it could be concluded that the proposed mathematical model successfully simulated the extraction of phenolics, flavonoids, and antioxidant activity from orange byproduct within the 5–25 °C temperature range, under mechanical stirring and acoustic assistance conditions.

### 3.3. Phenolic Composition of Orange Byproduct

Initial contents of flavonoids and phenolic acids compounds of orange byproduct are also shown in Table 1. Naringin content was the highest (41% of the six measured compounds), followed by neohesperidin and hesperidin (25% and 24% respectively). The sum of the contents of the three flavonoids was more than eight times higher than the content of the three phenolic acids. The phenolic acid contents represented only the 6% (ferulic acid), 4% (sinapic acid) and less than 1% (coumaric acid) of the six measured compounds. The sum of the contents of phenolics acids and flavonoid componend was lower than TPC and TFC, respectively. These results may be due to the use of the quercetin standard. This could react differently with the reagent respect to the flavonoids present in the samples. Similar results can be found in the studies of M’hiri et al. [37] and Herranz et al. [38].

**Figure 2:**
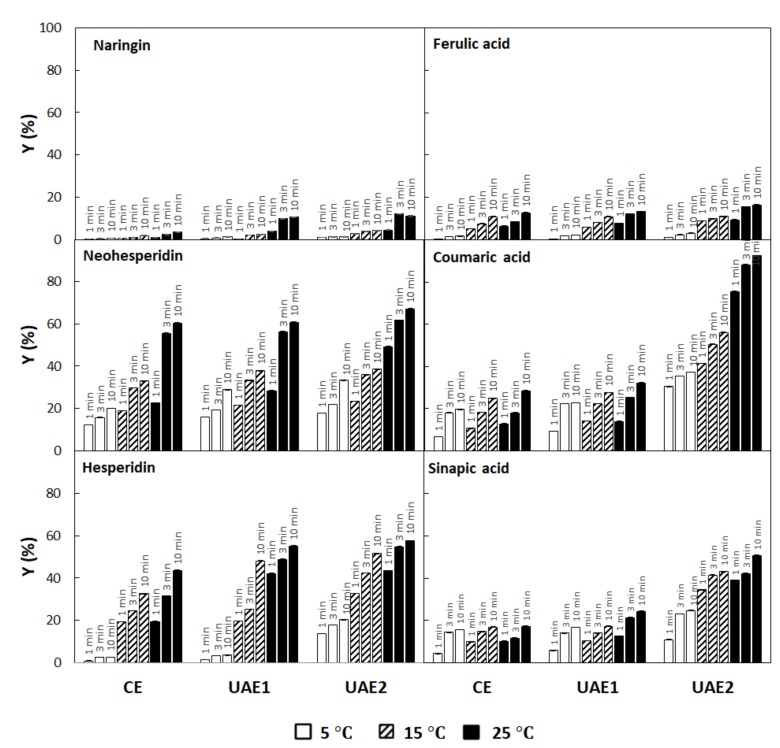
Extraction yields of flavonoid compounds (naringin, neohesperidin, and hesperidin) and phenolic acids ferulic acid, -coumaric acid, and sinapic acid) extracted during 1, 3, and 10 min from orange byproduct by conventional extraction (CE) and ultrasound-assisted extraction with two ultrasonic powers (UAE1 at 520 W/L; UAE2 at 790 W/L). Different lowercase letters for the same extraction process and same temperature indicates significant differences in time.

Figure 2 show the extraction yields of flavonoid compounds (naringin, hesperidin, and neohesperidin) and phenolic acids (ferulic acid, coumaric acid, and sinapic acid) at 1, 3, and 10 min, from orange byproduct obtained by conventional extraction (CE) and ultrasound-assisted extraction with two ultrasonic power (UAE1 at 520 W/L; UAE2 at 790 W/L). For all detected phenolic compounds under study, the extraction yields increased with both the extraction time and temperature. In agreement with our results, Ma et al. [39] reported similar percentages of extraction of coumaric acid (7.5–17.5 μg/g dm), sinapic acid (7.5–20.0 μg/g dm) and ferulic acid (50–220 μg/g dm) in Satsuma mandarin peel by ultrasound-assisted extraction (56 W) at 15 °C from 10 to 60 min. Moreover, Rodrigues and Pinto [40] reported that the extraction time significantly affected the yields of phenolic compounds from coconut shell powder.

Important increases in the extraction yields of each phenolic compound were observed with increasing temperature in both conventional and ultrasound assisted extractions, between 10% in cumaric acid and 1533% in hesperidin in conventional extractions, and between 102% in neohesperidin and 820% in narangin in ultrasound assisted extraction, when the temperature increased from 5 to 25 °C. The yield increase with the temperature was higher in neohesperidin, hesperidin, and ferulic acid extracted by the conventional method of agitation, but higher in naringin, courmic acid, and sinapic acid when extraction was assisted by ultrasound. Ragin et al. [41], Pinelo et al. [42] and Jokic et al. [43] also reported that the yields of phenolic compounds extracted from milled berries and grape pomace depended significantly on extraction temperature and time.

According to Figure 1, the maximum yields of TPC at 25 °C were of 56% in CE and 80% in UAE2. It can be observed in Figure 2 that the yields were very different among the different compounds. The use of ultrasound at 25 °C increased the yields of extraction of naringin by 216% and in ferulic acid by 29%. However, the extraction of these two components was low, most likely due to the dissolvent used to carry out the extraction (water), because both naringin and ferulic acid are low polar compounds compared to the other analyzed compounds.

Ultrasound promoted moderate extraction increases of 11.4% and 32% in neohesperidin and hesperidin, respectively, at 25 °C. For coumaric and sinapic acid extraction at the same temperature, however, the effect of ultrasound was exceptional, increasing their yield by 224% and 192%, respectively.

The extraction yields after UAE1 and UAE2 at 25 °C for 3 min, were significantly higher than those by conventional extraction at 25 °C for 10 min. These results showed that UAE promoted higher extraction efficiencies compared to conventional extraction. However, the extraction yields of phenolic compounds by UAE at 5 °C for 1 min were lower than those by conventional extraction at 25 °C for 1 min, indicating, in this case, that UAE at low temperature is not enough to increase the yields of phenolic compounds compared to conventional extraction. In line with the present research, Ma et al., [39] also showed that UAE at low temperature enhanced the extract yields of from Citrus *unshiu* peels.

A positive effect on the extraction yields of phenolic compounds was observed with the increase of the ultrasonic power. When ultrasonic power increased from 520 to 790 W/L, the yields of most phenolic compounds increased significantly (*p* < 0.05). For example, the yields of naringin, neohesperidin, hesperidin, coumaric acid, ferulic acid, and sinapic acid after 3 min at 15 °C increased 81%, 8%, 67%, 125%, 22%, and 194%, respectively, when ultrasonic power density increased from 520 to 790 W/L. The extraction yields of neohesperidin was much higher than those of the other compounds and exhibited a low increase from 520 to 790 W/L, most likely due to the fact that neohesperidin, among all detected phenolic compounds, is the most unstable molecule and could suffer from degradation reactions. However, the extraction yield of the sinapic acid did not exhibit a significant increase with the application of ultrasounds with a power density of 520 W/L compared to the CE extraction, but a considerable increase when ultrasonic power density increased from 520 to 790 W/L, most likely indicating that the extraction of sinapic acid requires a higher activation energy for its release. It has been reported that the effect of ultrasound variables on the extraction of multibioactive compounds from *Hypericum perforatum* L. and from Satsuma mandarin peel varied from one compound to another [39]. Different physicochemical properties of phenolic compounds may be responsible for the different extraction efficiencies under the same extraction conditions.

## 4. Conclusions

The experimental data discussed above clearly suggest that orange byproduct constitutes an important source of biocompounds, which could be extracted with high yields in water at low/moderate temperature (5–25 °C) when the extraction is assisted by ultrasound. The process is affected by both the temperature and the acoustic power density. The percentages of extracted compounds from the initial orange byproduct were of 83% of TPC, 75% of TFC and 95% of AA (ABTS assay) after 10 min of extraction at 25 °C and 790 W/L of acoustic power density, 28%, 44%, and 157% higher than the yields obtained at the same temperature by conventional extraction. Moreover, if extraction time at 25 °C and ultrasound at 790 W/L was reduced to 3 min, the TPC, TFC, and AA yields were also high (80%, 64%, and 95% respectively), 29%, 39%, and 197% higher than the yields obtained after 3 min of extraction at the same temperature by conventional extraction. Thus, the ultrasound-assisted extraction needed less extraction time and lower temperatures to obtain extracts with similar or even higher total phenol content and antioxidant activity than those obtained from conventional extraction.

The proposed Weibull model satisfactorily described both the mechanical and ultrasonic aqueous extraction kinetics of total phenol content, flavonoid total content and antioxidant activity within the temperature range from of 5 to 25 °C, the mean relative error of the simulation being of 3.1% ± 1.2%. The mathematical model allowed to conclude that the change extraction rate increased with both the temperature and the acoustic power density, the activation energy decreased with the application of ultrasound and the extraction rate decreased over extraction time in all cases, but sharply decreased when ultrasound were applied in the extraction of TFC.

Finally, by analyzing the phenolic composition of extracts, it was observed that different compounds were extracted in different yields. The neohesperidin, hesperidin, coumaric acid, and sinapic acid were those presenting the highest yields, especially when extraction was assisted by ultrasound (maximum yields of 67%, 58%, 92%, and 50%, respectively, after 10 min extraction at 25 °C and 790 W/L of acoustic power density). Extraction yields of naringin and ferulic acid were lower, most likely due to their lipophilic character (maximum yields of 11% and 16%, respectively, after 10 min extraction at 25 °C and 790 W/L of acoustic power density).

## Figures and Tables

**Table 1:** Total polyphenol (TPC) and flavonoid (TFC) contents, antioxidant activity (AA) (ABTS (2.2′-azino-bis(3-ethylbenzothiazoline-6-sulfonic acid), CUPRAC (cupric reducing antioxidant capacity), and FRAP (ferric reducing antioxidant power) assays) and contents of main flavonoids and phenolic acids of initial orange byproduct before extraction. Means and deviations.

		Mean ± Deviation
TPC (mg GAE/100 g dm)		1674 ± 15
TFC (mg catechin/100 g dm)		2849 ± 8
AA (mg Trolox/100 g dm)	ABTS assay	2810 ± 20
	CUPRAC assay	2760 ± 50
	FRAP assay	1820 ± 70
Flavonoids (mg/100 g dm)	Naringin	1100 ± 40
	Neohesperidin	660 ± 50
	Hesperidin	660 ± 11
Phenolic acids (mg/100 g dm)	Coumaric acid	25 ± 2
	Ferulic acid	158 ± 13
	Sinapic acid	95 ± 3

**Table 2:** Equilibrium extraction yields (Y_eq_, %) of total polyphenol (TPC) and total flavonoid (TFC) contents, and antioxidant activity (AA) (ABTS assay) for mechanical agitation (CE (conventional extraction) at 80 rpm) and acoustically assisted (UAE1 at 520 W/L; UAE2 at 790 W/L) extraction and their observed temperature dependence within the temperature range 5–25 °C.

	Y_eq_ (%)		
	CE	UAE1	UAE2
TPC	67.3 ± 1.1	74.7 ± 1.8	83.0 ± 0.4
	T dependence not observed	T dependence not observed	T dependence not observed
TFC	Y_eq_ = 0.301 T (°C) + 48.74	Y_eq_ = 1.398 T (°C) + 46.28	Y_eq_ = 0.977 T (°C) + 55.64
	*r^2^* = 0.993	*r^2^* = 0.988	*r^2^* = 0.989
AA (ABTS assay)	Y_eq_ = 0.063 T (°C) + 36.17	Y_eq_ = 0.153 T (°C) + 68.35	Y_eq_ = 0.317 T (°C) + 86.92
	*r^2^* = 0.982	*r^2^* = 0.989	*r^2^* = 0.990

**Table 3:** Identified parameters (α_0_, Ea and β) for the proposed Weibull model to simulate the extraction yield curves of total polyphenol (TPC) and total flavonoid (TFC) contents, and antioxidant activity (AA) (ABTS assay) for mechanical agitation (CE at 80 rpm) and acoustically assisted (UAE1 at 520 W/L; UAE2 at 790 W/L) extraction; together with the confidence interval and standard error (SE) associated to each parameter.

		CE			UAE1			UAE2		
		Value	Conficence Interval	SE	Value	Conficence Interval	SE	Value	Conficence Interval	SE
TPC	α_0_ (s^−1^)	4.09 × 10^−10^	(3.14 × 10^−10^, 5.04 × 10^−10^)	9.9	5.09 × 10^−7^	(4.01 × 10^−7^, 6.17 × 10^−7^)	0.4	1.69 × 10^−3^	(−7.36 × 10^−3^, 1.07 × 10^−2^)	0.3
	E_a_ (J/mol)	62948.8	(46732, 79165)	5727	43058.2	(32556.6, 53559.8)	1783	23103.4	(10666.3, 35552.5)	4394
	β	0.609	(0.467, 0.751)	0.1	0.529	(0.438, 0.620)	0.7	0.618	(0.457, 0.780)	0.1
TFC	α_0_ (s^−1^)	1.83 × 10^−7^	(1.37 × 10^−7^, 2.28 × 10^−7^)	0.49	5.76 × 10^−2^	(4.40 × 10^−2^, 4.11 × 10^−2^)	0.7	5.77 × 10^−2^	(4.3 × 10^−2^, 7.2 × 10^−3^)	0.4
	E_a_ (J/mol)	48065.9	(35472, 60659)	4147	15518.9	(11564, 19473)	170	15518.8	(11429, 19608.7)	4039
	β	0.460	(0.356, 0.564)	0.1	0.025	(0.019, 0.031)	0.8	0.009	(0.007, 0.011)	0.1
AA (ABTS)	α_0_ (s^−1^)	5.31 × 10^−11^	(4.17 × 10^−11^, 6.45 × 10^−11^)	0.49	1.28 × 10^−7^	(9.88 × 10^−8^, 1.56 × 10^−7^)	0.6	5.01 × 10^−7^	(3.9 × 10^−7^, 6.1 × 10^−7^)	0.7
	E_a_ (J/mol)	66300.2	(48057, 84543)	4816	46010.7	(34667, 57355)	159	41050.3	(30342, 51758)	2305
	β	0.371	(0.285, 0.457)	0.1	0.501	(0.357, 0.625)	0.8	0.502	(0.381, 0.622)	0.2

**Table 4:** Mean relative error (MRE) estimated by comparison of experimental and simulated extraction yield curves of total phenolic (TPC) and total flavonoid (TFC) contents, and antioxidant activity (AA) (ABTS assay) from orange byproduct by mechanical agitation (CE at 80 rpm) and acoustically assisted (UAE1 at 520 W/L; UAE2 at 790 W/L) extraction.

	MRE (%)		
	TPC	TFC	AA (ABTS)
CE	4.6	3.4	5.0
UAE1	3.7	2.6	2.7
UAE2	1.6	2.3	1.8
Average	3.3 ± 1.6	2.8 ± 0.6	3.1 ± 1.7

## Abbreviations

AA	antioxidant activity	mg trolox/g dm
ABTS	2.2′-azino-bis(3-ethylbenzothiazoline-6-sulfonic acid	mg trolox/g dm
C	concentration	g/g dm or g/100 g dm
C_max_	initial concentration	g/g dm or g/100 g dm
C_eq_	equilibrium concentration	g/g dm or g/100 g dm
C_p_	specific heat	J/Kg °C
CI	confidence intervals	
CUPRAC	Cupric reducing antioxidant capacity	mg trolox/g dm
dm	dry matter	
E_a_	activation energy	J/mol
FRAP	Ferric reducing antioxidant power	mg trolox/g dm
GAE	gallic acid equivalent	
m	mass	kg
MRE	mean relative error	(%)
n	number of observations	
P	ultrasound power	W
SE	standard error	
t	time	s
T	temperature	°C
TFC	total flavonoid content	mg catechin/100 g dm
TPC	total polyphenol content	mg GAE/g dm
UAE	ultrasound assisted extraction	
Y	extraction yield	(%)
Y_calc_	calculated extraction yield	(%)
Y_eq_	equilibrium extraction yield	(%)
Y_exp_	experimental extraction yield	(%)
α	kinetic reaction constant of the Weibull model	s^−1^
α_o_	pre-exponential factor in Arrhenius equation	s^−1^
β	shape parameter of the Weibull model	
